# Alien Hand Syndrome Unveiled in a Patient With Right Middle Cerebral Artery Stroke

**DOI:** 10.7759/cureus.52552

**Published:** 2024-01-19

**Authors:** Ghada Rashwan, Sara Elagha, Tahani Aldaham, Liza Thomas

**Affiliations:** 1 Internal Medicine, Rashid Hospital, Dubai Academic Health Corporation, Dubai, ARE

**Keywords:** right middle cerebral artery stroke, involuntary limb movement, alien limb syndrome, posterior variant, alien hand syndrome

## Abstract

Alien hand syndrome (AHS) is a rare but significant disorder, characterized by involuntary and often complex movements of one’s hand. These movements seem to occur independently and unconsciously, separate from a person's intended actions.

We report a case of a 70-year-old male who presented to the emergency department with left sided hemiplegia following a stroke in the right middle cerebral artery (MCA) that affected the right temporal, parietal and internal capsule region. Upon additional inquiry, the patient reported experiencing involuntary, abrupt movements in his left upper and lower limbs particularly while attempting voluntary movements. As per the patient, these symptoms occurred prior to the onset of limb weakness. The diagnosis of AHS was made after excluding other differentials based on the clinical, metabolic and radiological picture.

Our patient exhibited unique symptoms and had a different presentation from that mentioned in the literature, as the onset of symptoms preceded the development of limb weakness typically associated with stroke. Involvement of the upper and lower limb and onset of symptoms prior to limb weakness make this case exceptionally rare. Moreover, acknowledging alien hand/limb syndrome as a distinct condition, separate from the broad category of post-stroke movement disorder, carries significant implications for both the management and prognosis of affected individuals.

## Introduction

Alien hand syndrome (AHS) is a rare neurological syndrome characterized by un-willed, uncontrollable but seemingly purposeful movements of the limbs. Patients with AHS experience involuntary movements of the affected limb, which can feel like it is performing actions without their conscious control or being controlled by an external force; hence the name ‘alien hand’ [1]. Due to its rarity, the exact prevalence is unknown but is estimated to be low.

AHS was first described in 1908 by Goldstein after a patient developed involuntary limb movements after a stroke. He described it as ‘a type of apraxia with a feeling of estrangement between the patient and his hand’ [2]. However, it was only until 1972 that Brion and Jedynak introduced the term ‘alien hand syndrome’ after observing three patients with callosal tumors that were unable to recognize their own hands [2].

Although no clear criteria exists to diagnose AHS, Doody and Jankovic described it as the following: failure to recognize ownership of the limb when visual clues are removed, a feeling that one body part is foreign, personification of the affected body part, and autonomous activity which is perceived as outside voluntary control [3]. 

AHS is associated with multiple pre-existing conditions such as stroke, multiple sclerosis, neurosurgical procedures, neurodegenerative diseases such as Creutzfeldt-Jakob disease (CJD), corticobasal syndrome (CBS), etc. with stroke being the most common pre-existing condition [4]. 

AHS presents with several types of abnormal movements according to the region of the brain that is affected. These types include: 1) diagnostic dyspraxia, when one hand performs functions contrary to the other hand; 2) alien hand sign, which is the feeling that the hand does not belong to them; 3) syndrome of anarchic hand, when the hand performs goal-directed activity not under the will of the person; 4) levitating hand, when the hand levitates without voluntary movement by the person; and 5) self-groping/self-oppositional behavior [3,5]. 

This case is of great significance due to its infrequent occurrence. While AHS has been documented in the literature, the presence of alien hand and limb syndrome, as observed in our case, is exceptionally uncommon, with only a handful of similar cases reported in existing literature to date [6]. 

## Case presentation

A 70-year-old male, with a history of uncontrolled diabetes, presented to the emergency department with complaints of left-sided upper and lower limb weakness for around five hours, associated with reduced vision in the left eye. On further history from the patient’s family, it was noted that he had been having abnormal movements in the left arm for three to four days prior to the onset of weakness. These movements were described as rapid and abrupt, causing his arm to move uncontrollably, mainly upwards, when trying to reach for an item. The family added that he was unable to control these movements, which affected both his left arm and left leg. Furthermore, his wife stated that during the night, these movements would result in unintentional kicking or punching her during his sleep. Upon taking history from the patient himself, he confirmed that the movements have been occurring for the past week and he was unable to control them. He stated that he noticed these movements would happen throughout the day against his will, regardless of whether he was trying to move his limb or not. The patient demonstrated how his arm would move in different directions whenever he would try to intentionally grasp an object or perform any movements.

CT brain done in the emergency department showed a well-established right middle cerebral artery (MCA) stroke. Angiography showed occlusion of the inferior branch of M2 segment of the right MCA. 

Based on these symptoms and CT findings, the patient was admitted under the Internal Medicine department as a case of acute ischemic stroke secondary to infarction of the right MCA affecting the temporoparietal and internal capsule region. In addition, transthoracic and transesophageal echocardiogram were done to rule out cardioembolic etiology, both of which were negative. Brain MRI could not be done due to the metal prosthetics in patients’ knees that were incompatible with the MRI machine. 

During his hospital stay, the neurology team was consulted for further assessment and management of his abnormal limb movements. As per their assessment, he was diagnosed as a case of post-stroke movement disorder and was started on a low dose of clonazepam (0.5mg twice daily) and kept under observation. In addition, the neurology team advised to perform an electroencephalogram (EEG) to rule out seizures, which was reported to be normal. On day three of admission, a subsequent evaluation revealed no signs of progression or deterioration after initiation of treatment. On the 13th day after admission, the patient exhibited clinical deterioration which was due to hemorrhagic transformation of the MCA stroke. Following this, the patient's jerky movements slightly improved, possibly as a result of the declining power following the hemorrhagic transformation.

On examination upon admission, his vitals were within normal range except for high blood pressure measuring 185/90 mmHg. On general examination he was conscious, alert and oriented to time, place and person. CNS examination revealed a Glasgow Coma Scale (GCS) score of 15/15. Motor power of the right upper and lower limb was found to be 5/5 and left upper and lower limb was 4+/5. Sensory examination showed loss of touch and pin-prick sensation in both left upper and lower limb, and loss of proprioception on the left side. The patient also had left-sided neglect. Right side showed no changes. Cranial nerves examination revealed left sided hemianopia and seventh cranial nerve palsy, with right sided facial deviation and decreased sensation on the left side of the face. Cerebellar examination showed overshooting of left upper limb on finger to nose test. Gait was not assessed. Following the hemorrhagic transformation, motor power reduced to 0/5 in the left upper and lower limb.

The patient was released from the hospital 17 days post admission. Upon discharge, power in the left upper limb improved to 2/5. The patient was advised to continue physiotherapy and clonazepam and a follow up appointment in the neurology clinic was given. 

After a lapse of four months, the patient provided an update, indicating that the symptoms had completely resolved; however, the patient was uncertain about the precise timeline of this resolution. Notably, the patient ceased using clonazepam approximately one month after discharge. 

**Figure 1:**
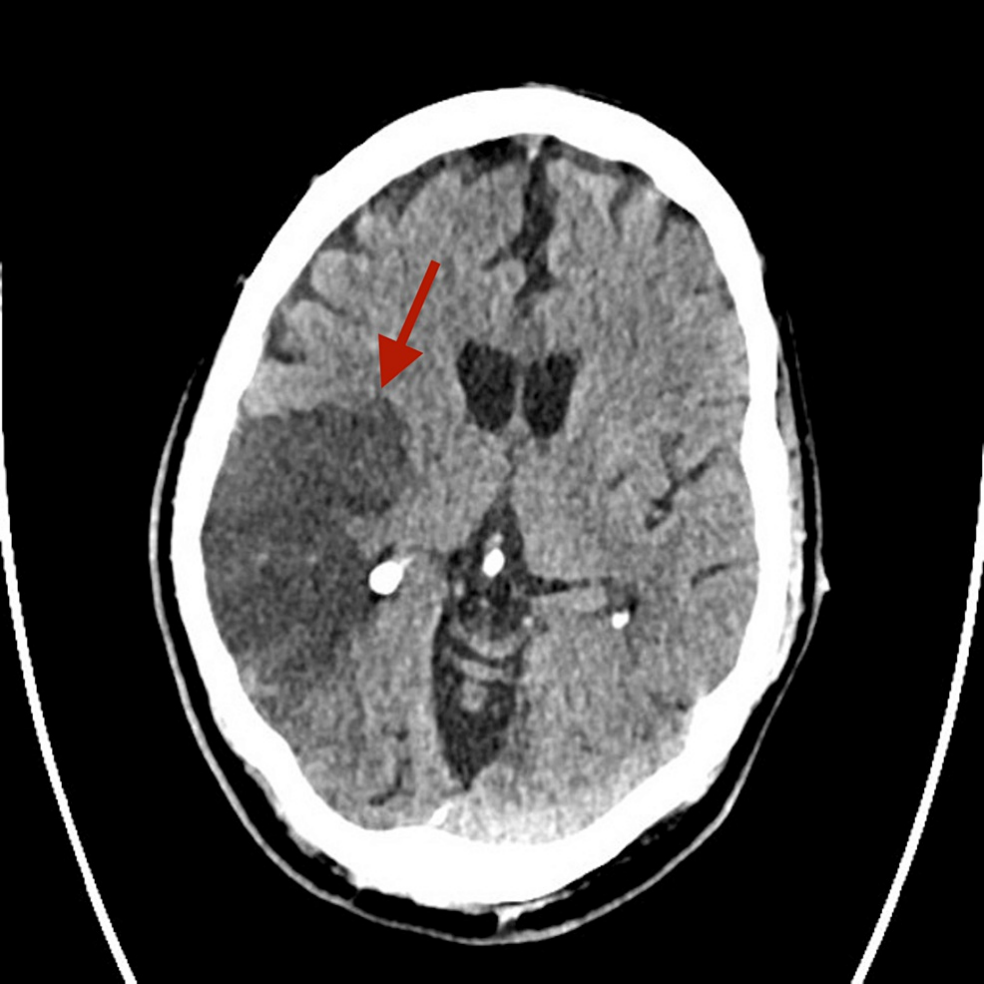
Initial non-contrast CT scan on hospital admission revealing an ischemic stroke in the right middle cerebral artery (MCA) territory (temporo-parietal region).

**Figure 2:**
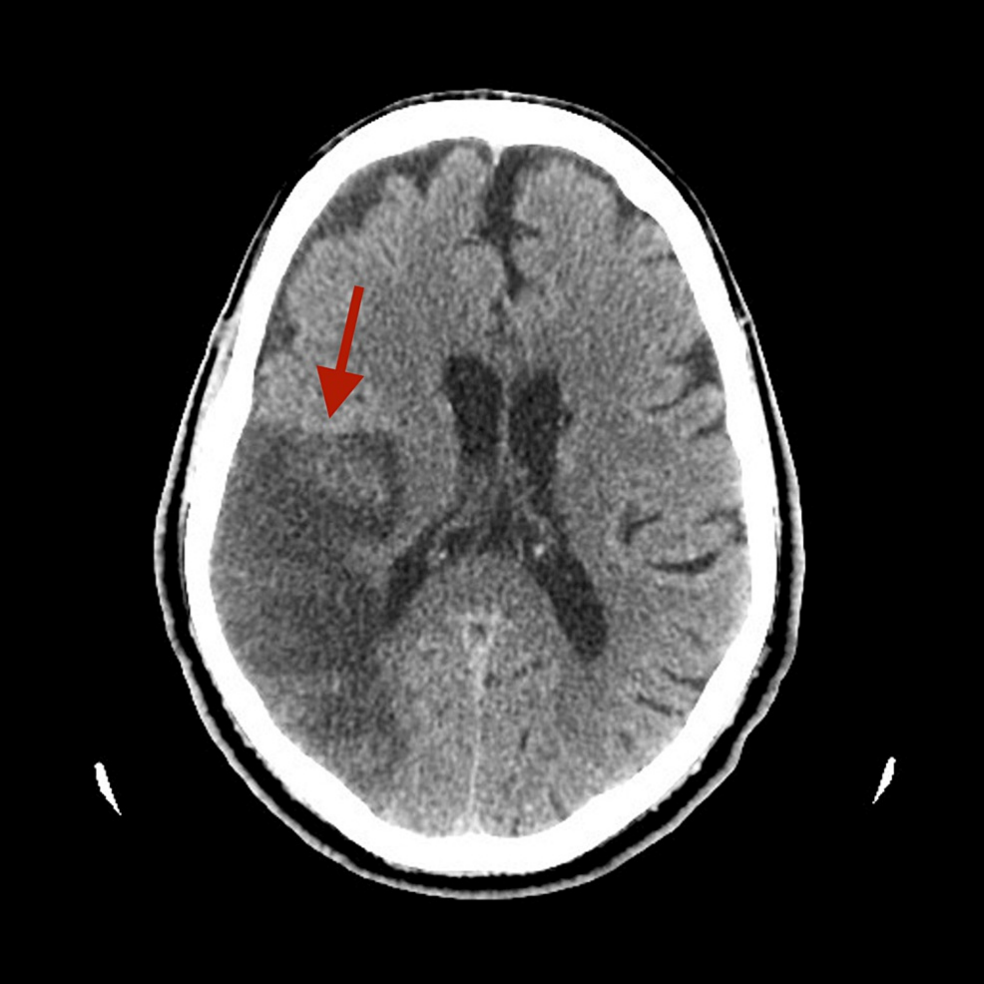
Follow-up non-contrast CT scan on day four revealing expansion of the right middle cerebral artery (MCA) stroke.

**Figure 3:**
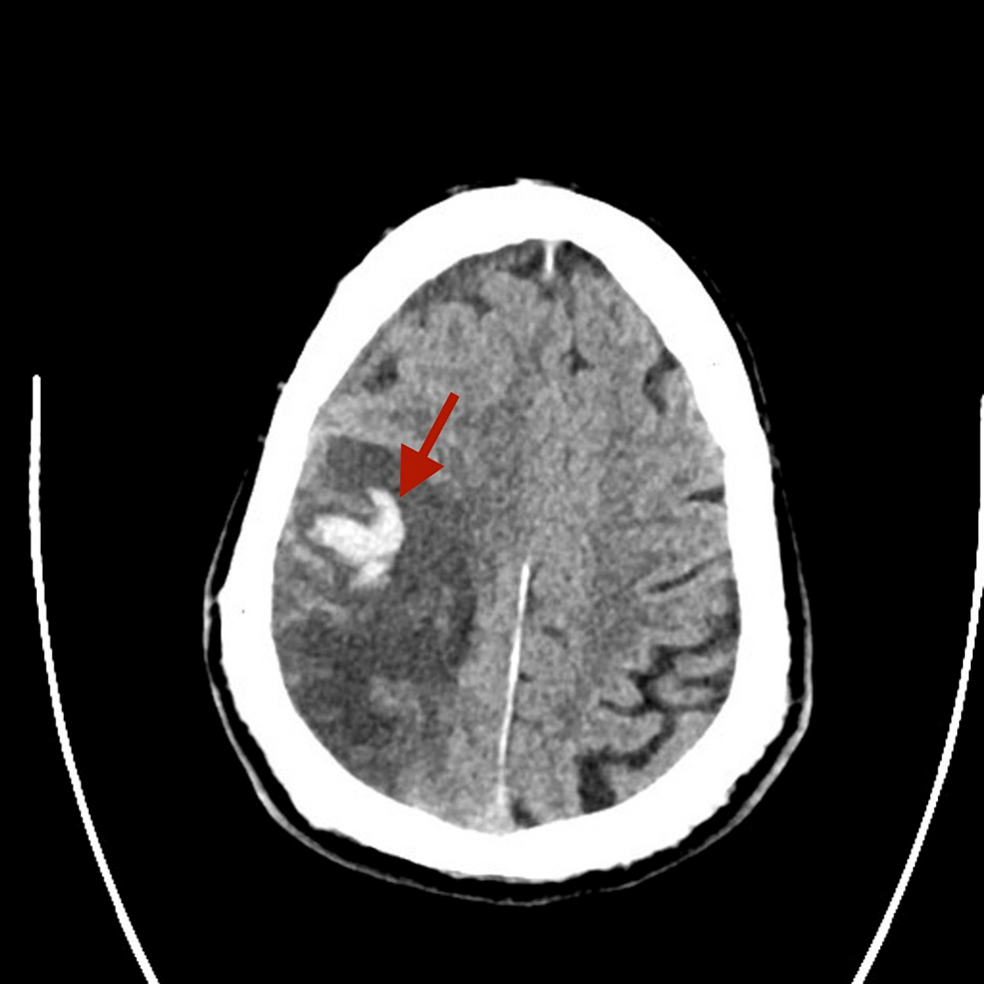
Repeat non-contrast CT brain done on day 13 post clinical deterioration revealing hemorrhagic transformation of the right middle cerebral artery (MCA) stroke.

## Discussion

AHS encompasses an intriguing yet poorly understood group of movement disorders. The literature identifies two distinct forms of AHS: the anterior variant, which includes frontal and callosal subtypes, and the posterior variant. Each type presents with unique clinical features and occurs due to lesions in different anatomical regions [5]. However, overlap between subtypes is common [7].

The frontal form of this condition typically affects the dominant hand and is characterized by impulsive groping and grasping behavior. It is caused by damage to the supplementary motor area (SMA), anterior cingulate gyrus, and medial prefrontal cortex of the dominant hemisphere, with or without callosal damage. The leading cause of the frontal variant is a stroke in the anterior communicating artery territory [8]. Conversely, the callosal form affects the non-dominant hand and is characterized by symptoms of callosal disconnection syndrome such as apraxia, neglect, and agraphia [7]. This is primarily caused by injury to the corpus callosum resulting from neurosurgical procedures, callosal hemorrhages or infarcts [8,9]. In addition, intermanual conflict is a common feature of the callosal variant, which involves opposing purposeful movements of the hands which may appear as ‘hands fighting each other’. This is commonly observed in those with lesions in the anterior third of the rostrum [8,10].

Finally, the posterior variant mainly affects the non dominant hand and is associated with symptoms such as limb levitation, non-purposeful movement, visual or sensory neglect, and is primarily caused by lesions in the thalamus, occipital and posterolateral parietal lobe [7]. Common etiologies of the posterior variant include neurodegenerative diseases and strokes in the parietal lobe or posterior cerebral artery territory [7]. 

Based on the above classification, our patient met the criteria for posterior AHS as he had a stroke affecting the parietal lobe and had symptoms of limb levitation and neglect.

Our patient exhibited unique symptoms and had a different presentation from that mentioned in the literature as the onset of AHS symptoms preceded the development of limb weakness typically associated with stroke. This peculiar presentation led to oversight of the patient’s condition and was assumed by the patients’ family that these abnormal limb movements were voluntary. Kelly Le et al. reported a comparable case in which a patient displayed AHS symptoms three hours prior to experiencing limb weakness, and was subsequently diagnosed with a right-sided stroke affecting the parietal and temporal region. The symptoms in this case resolved spontaneously one day after admission [11]. 

In addition, our patient also exhibited abnormal movements of the left lower limb. According to a study published in 2021, there have only been six reported cases of alien leg syndrome so far, with the underlying causes being stroke, CBS and CJD [6]. Ghazal Haeri et al. reported two cases of alien leg syndrome without upper limb involvement, both due to underlying corticobasal syndrome [6]. Banshi Lal Kumawat et al. reported a case of alien hand and leg as a presenting symptom of sporadic CJD, the patient rapidly deteriorated and developed truncal ataxia and cognitive decline within 15 days of admission [12].

In our case the symptoms persisted throughout hospital stay, lasting for one to two minutes, despite the patient receiving a total daily dose of 1 mg of clonazepam. However, the patient remained stable throughout, and it was only on the 13th day of admission where deterioration occurred. This deterioration was attributed to hemorrhagic transformation of the ischemic stroke and was not associated with the alien limb phenomenon. Following this, the patient’s abnormal arm movements showed partial improvement which was likely due to exacerbation of the patient’s limb weakness rather than a direct improvement in the underlying condition. 

The underlying pathophysiology of AHS plays a crucial role in understanding the mechanism and clinical manifestations. The frontal subtype is associated with damage to the frontal lobes particularly the SMA and the premotor cortex. Damage to the SMA possibly results in release of the inhibitory motor control on the ipsilateral side of the brain, thereby resulting in involuntary limb movements of the contralateral side [13,14]. The callosal subtype is associated with damage to the cingulate gyrus and the corpus callosum [2]. Damage to this area can cause involuntary grasping movements of the opposite arm, can affect how strong or forceful the grasping movements are, and contributes to a feeling of unfamiliarity with the affected limb [15]. Additionally, hand movements are coordinated through transfer of information from bilateral frontal lobes through the anterior corpus callosum. Studies have shown that if there is a lesion in the corpus callosum, the intended hand may not be able to execute the action while the opposite hand can [16]. The rarer type, the posterior variant, is mainly associated with cortical parietal pathology. The parietal cortex is important for visuospatial orientation, thus, lesions in this area result in proprioceptive errors and inability to perform goal directed activity [13]. The superior and inferior parietal lobules work together and with other centers in the brain to ensure coordinated limb movements. Disruption of this network leads to involuntary limb movements and patients feel as if their limb has a mind of its own [17]. 

The posterior variant of AHS is frequently observed in individuals with CBS. According to a study conducted at Mayo Clinic between 1996 and 2011, out of 150 patients with AHS, 108 had underlying CBS [18]. 

Furthermore, an EEG was conducted to rule out the possibility of a seizure disorder as an important differential diagnosis for these movements, which was normal. Additionally, it is worth noting that the patient was fully conscious and aware during the movements. Although other movement disorders, such as chorea and hemiballismus were considered, the patient's arm movements and radiological findings did not align with the typical presentation of these disorders. Distinguishing movement disorders and seizures can often be quite challenging as both may exhibit similar symptoms of involuntary, uncontrollable, jerky limb movements. However, these conditions have different causes, mechanisms and treatment, therefore, an EEG and a detailed medical history is quite useful to help differentiate between them.

Moreover, it is crucial to consider another diagnosis in light of our patients' history of uncontrolled diabetes mellitus, namely hyperglycemia induced hemiballismus/hemichorea. Nevertheless, upon admission, our patient had a blood sugar of 247mg/dl and a plasma osmolality of 285mOsmol/kg, effectively eliminating hyperosmolar hyperglycemic state. In addition, the lesion typically associated with hyperglycemia-induced hemiballsimus/hemichorea occurs in the striatum basal ganglia, which was not the case in our patient. 

Finally, despite several theories proposed, the exact pathophysiology of posterior AHS remains unknown [19]. At present, no definitive treatment for the condition exists; however, trials have shown promising results with medical therapy, including clonazepam and botulinum toxin, as well as rehabilitation therapies like mirror therapy and spatial recognition therapy, leading to successful recovery in some cases [7]. 

## Conclusions

To conclude, despite its rarity, AHS can be a debilitating condition that causes significant distress to those who experience it. A deeper understanding of this condition is crucial for delivering improved and targeted care, particularly in cases with novel manifestations. This highlights the importance of continuous research and education to effectively deal with the challenges presented by AHS. 
